# COVID-19 in the autopsy room–requirements, safety, recommendations and pathological findings

**DOI:** 10.1007/s12024-020-00341-1

**Published:** 2021-01-04

**Authors:** Jacek Baj, Marzanna Ciesielka, Grzegorz Buszewicz, Ryszard Maciejewski, Barbara Budzyńska, Piotr Listos, Grzegorz Teresiński

**Affiliations:** 1 Chair and Department of Anatomy, Medical University of Lublin, 20-090 Lublin, Poland; 2 Chair and Department of Forensic Medicine, Medical University of Lublin, 20-090 Lublin, Poland; 3 Independent Laboratory of Behavioral Studies, Medical University of Lublin, 20-090 Lublin, Poland; 4 Department and Clinic of Animal Internal Diseases, Sub-Department of Pathomorphology and ForensicMedicine, Faculty of Veterinary Medicine, University of Life Sciences in Lublin, Lubin, Poland

**Keywords:** COVID-19, Autopsy, Forensic, Postmortem computed tomography, Burial

## Abstract

Modern technologies enable the exchange of information about the expansion of severe acute respiratory syndrome coronavirus 2 (SARS-CoV-2) infection and the continually increasing number of the coronavirus disease 2019 (COVID-19) cases almost in real time. The gravity of a current epidemiological situation is represented by the mortality rates, which are scrupulously updated daily. Performing autopsies on patients with either suspected or confirmed COVID-19 is of high importance since these might not only improve clinical management but also reduce the risk of SARS-CoV-2 infection expansion. The following paper aimed to present the most crucial aspects of SARS-CoV-2 infection from the point of view of forensic experts and pathologists, recommendations and safety precautions regarding autopsies, autopsy room requirements, possible techniques, examinations used for effective viral detection, recommendations regarding burials, and gross and microscopic pathological findings of the deceased who died due to SARS-CoV-2 infection. Autopsies remain the gold standard for determining the cause of death. Therefore, it would be beneficial to perform autopsies on patients with both suspected and confirmed COVID-19, especially those with coexisting comorbidities.

## Introduction

The COVID-19 pandemic, also known as ‘coronavirus disease of 2019′, has become a worldwide concern due to severe acute respiratory syndrome with coronavirus 2 (SARS-CoV-2), going far beyond the actual capabilities of local healthcare systems [1–3]. A global shortage of diagnostic tests that many countries faced once the World Health Organization (WHO) declared that the outbreak of the pandemic was overwhelming and the limited capabilities to perform such tests by the medical staff overstretched the potential of health care systems worldwide. At the same time, researchers and clinicians continuously sought the most effective drugs against SARS-CoV-2 [4–6].

Diagnostic procedures depend on the degree of disease severity, and they are different for asymptomatic but suspected patients and for those who present with specific clinical manifestations. In addition, tests for influenza or other pathogens can cause respiratory infections, and specific laboratory tests (complete blood count (CBC), arterial blood gas assessments) and radiological tests (primarily computed tomography (CT), RTG, or ultrasonography (USG)) are performed on a regular basis. The three diagnostic strategies – genetic tests, antigen tests, and antibody tests – currently constitute a routine laboratory diagnosis of SARS-CoV-2 infection. Reverse transcription real-time polymerase chain reaction (rRT-PCR) is currently a basic technique that enables a relatively quick detection of SARS-CoV-2 infection [7].

SARS-CoV-2, recently categorized as an HG3 organism, constitutes a significant risk for employees who face infected patients [8–10]. The emergency measures that aim to minimize the epidemiological risk of SARS-CoV-2 spread must be maintained at all levels of the healthcare system. Therefore, it is of great importance to implement strict requirements concerning the management of the deceased who died because of COVID-19. Regarding the high risk of infection of medical personnel, primary care staff, morgue staff, and funeral and transportation agency workers, the implementation of specific safety requirements appears to be crucial. To date, numerous scientific organizations and societies worldwide have prepared specific recommendations regarding postmortem examinations and autopsies. These are highly differentiated and have been implemented with different aims and scopes – from underestimating the safety requirements to extreme precautions that aim to minimize the risk of infection.

Although the clinical features and radiological findings of SARS-CoV-2 infection have been well described so far, the early detection of potential SARS-CoV-2 infection would significantly minimize the epidemiological risk. Invariably, a classical autopsy remains the gold standard for determining the causes and mechanisms of death, as it also allows a gross examination and microscopic examination [11]. It is crucial to perform an autopsy to provide insight into the possible mechanisms of SARS-CoV-2 action and the extent of organ involvement targeted by the virus [12].

Safety requirements and proper techniques for the autopsy would significantly improve the management of a continually increasing number of deceased individuals with COVID-19. In addition, a full autopsy with both macroscopic and microscopic examinations would provide insight into manifestations of SARS-CoV-2 infection that might not be easily detected ante mortem. According to the WHO, bodies are generally not infectious except for patients with hemorrhagic fever (such as Ebola or Marburg) and cholera; however, there is no evidence of whether the deceased with COVID-19 might constitute a possible source of infection [13]. Furthermore, a history of no exposure does not completely guarantee that the deceased is not infected with SARS-CoV-2. Therefore, the early detection of an infection in a deceased individual who was suspected of having COVID-19 would significantly decrease the epidemiological risk associated with potential exposure to medical personnel and the family and acquaintances of the deceased and enable an efficient identification of a possible source of infection. Therefore, it is crucial to provide security measures that minimize the risk of exposure and at the same time respect the rights of the families of the deceased while providing the option to investigate the actual cause of death.

## Recommendations during COVID-19 autopsies

### Chinese recommendations – Zhejiang school of medicine

According to Chinese recommendations, there are three types of protection levels whose application depends on the possible risk of infection during the autopsy of patients with either suspected or confirmed COVID-19 (Table 1) [14]. While performing an autopsy, degree III protection is required. While handling the bodies of deceased patients (with either suspected or confirmed COVID-19), personal protective equipment (PPE) should include a work uniform, a disposable surgical cap, gloves, an additional pair of long-sleeved thick rubber gloves, a disposable medical uniform, an N95 respirator face mask or a powered air-purifying respirator with a face shield, work shoes/rubber boots with waterproof covers, and a waterproof apron and isolation gown. Preparation of the body requires filling all possible body openings (mouth, nose, ears, tracheotomy openings, and anus) with cotton balls or gauze dripped into either 3000-5000 mg/L chlorine-containing disinfectant or 0.5% peroxyacetic acid. Furthermore, the body should be packed in a double-layer cloth sheet dipped into disinfectant and then packed into an additional double-layer sheet soaked with disinfectant (containing chlorine).

**Table 1:** Protection degree and recommended personal protective equipment required during chosen procedures

Protection degree	Personal Protective Equipment	Application
Degree I	Disposable surgical cap Disposable surgical mask Disposable latex gloves Work uniform Disposable isolation clothing might be applied in necessary circumstances	General outpatient department Pre-examination with triage
Degree II	Disposable surgical cap N95 respirator face mask Disposable latex gloves Goggles Work uniform Disposable medical uniform	Fever outpatient department Isolation wards in hospitals Non-respiratory examination of suspected/confirmed cases Radiological examination of suspected/confirmed cases Cleaning of the tools that were used while handling with suspected /confirmed cases
Degree III	Disposable surgical cap N95 respirator face mask Disposable latex gloves Respiratory protective devices (full-face) or powered air-purifying respirator Work uniform Disposable medical uniform	During the following procedures: tracheal intubation, tracheotomy, bronchofibroscope, gastroenterological endoscopy (the procedures with the increased risk of subsequent infection of medical staff) Carrying out operations of suspected/confirmed cases Performing an autopsy in suspected/confirmed cases In cases when staff carries out NAT for COVID-19

### World health organization

The World Health Organization (WHO) announced that the autopsy of people who died due to COVID-19 should follow the same procedures and standards as in patients who died due to any other acute respiratory illness. Since the lungs of patients with pandemic influenza might be infectious when handled improperly, additional respiratory protection is recommended during the autopsy of patients with suspected and confirmed COVID-19 [15]. Furthermore, healthcare facilities must ensure that they provide appropriate safety measures to those who perform an autopsy on patients with either confirmed or suspected COVID-19. Appropriate PPE, such as scrub suits, long-sleeved fluid-resistant gowns, gloves, face shields or goggles, and boots, which are required during an autopsy, should be provided. While performing the autopsy, face shields are more strongly recommended than goggles; there is a need to wear either two pairs of gloves or one pair of special protective gloves during autopsies. While performing aerosol-generating procedures, N95, FFP2, FFP3 masks or their equivalents should be worn [16]. The number of personnel involved in the autopsy should be reduced to a minimum, and the autopsy room should be ventilated sufficiently. According to the WHO, individuals who have died because of COVID-19 can be either buried or cremated. Families and friends are allowed to see the body before the burial, however, without physical contact.

### The royal college of pathologists of the United Kingdom

According to The Royal College of Pathologists, in a deceased individual with SARS-CoV-2 infection, both external and internal examinations are equally important [8]. The universal precautions during autopsy include the use of round-ended scissors and PM40 blades and operating on one body cavity at a time. During the autopsy, PPE should include a surgical scrub suit, a hat, a visor, respiratory protection (either a surgical mask or an FFP3 mask), a waterproof gown protecting the whole body, a plastic apron, rubber boots with metal-protected toecaps, and gloves [17]. Since surgical masks do not provide sufficient protection, FFP3 masks are recommended during the autopsies of patients with suspected COVID-19. One hundred percent protection would be provided while using full-body suits with air-purifying respirators with air filters. When sawing the skull, an oscillator saw with suction extraction of aerosol in the bone should be used; when it is not available, a handsaw can be used.

The samples collected for further diagnosis should include those from the upper respiratory tract (nose and throat swabs) and the lower respiratory tract (sputum, bronchoalveolar lavage), as well as blood for serology [18, 19]. In addition, it is recommended that swabs and tissue samples from the respiratory tract be collected during the autopsy. A histological assessment should also be provided in addition to a microbiological/viral assessment. Among the abovementioned samples, nasal swabs are the most preferred to confirm suspected cases of COVID-19. Blood, urine, and cerebrospinal fluid should be collected before the body cavity is opened under sterile conditions to minimize the risk of contamination.

### Centers for disease control and prevention

While conducting an autopsy, standard contact and airborne precautions should be followed [20]. Furthermore, it is recommended to limit the number of personnel in the room where the autopsy is performed. All the autopsies of COVID-19 patients should be performed in Airborne Infection Isolation Rooms (AIIRs). PPE should include fluid-resistant gowns, a waterproof apron, goggles or a face shield, double surgical gloves with a layer of synthetic mesh gloves, and an N95 respirator [21].

The Centers for Disease Control and Prevention (CDC) emphasize that the collection of samples from the deceased differs depending on whether the case is confirmed and whether an autopsy will be performed [22]. When an autopsy of an individual with suspected COVID-19 is to be performed, it is recommended that nasopharyngeal swabs, swabs from both lungs and swabs from each lung to test for the presence of other pathogens, as well as tissue samples (fixed in formalin) from the lungs, upper airways, and other major organs, are collected [23]. In patients with suspected COVID-19 who do not undergo an autopsy, it is recommended that nasopharyngeal swabs and other samples be collected to detect other potential pathogens. An autopsy of a confirmed case of COVID-19 requires the collection of all the above-mentioned samples. The recommended sample collection sites include the trachea (proximal and distal), central lung with segmental bronchi and right and left primary bronchi, as well as parenchyma from both lungs. There is no need to collect samples in a patient with confirmed COVID-19 antemortem. Samples should be stored at 2–8 °C for up to 72 h after collection; in cases of delayed testing, the samples should be stored at a maximum temperature of -70 °C.

### College of American pathologists

The College of American Pathologists (CAP) recommends that the autopsies of patients with either confirmed or suspected COVID-19 be performed only by personnel who are trained in performing autopsies on deceased individuals with emerging infectious diseases using all the necessary PPE [24]. The CAP Autopsy Committee supports the guidelines regarding PPE and facilitates those used during the autopsy recommended by the CDC [25]. According to the CAP, powered air-purifying respirators can be applied as an alternative to N95 masks [25]. The decision to perform an autopsy in suspected COVID-19 cases should be made after consulting the head of the pathology department. The CDC guidelines regarding sample collection are supported by the CAP Autopsy Committee [8]. In patients with acute kidney injury or myocardial injury, it is recommended that renal and myocardial tissues are sampled [26, 27]. Furthermore, the CAP Autopsy Committee encourages forensic medicine departments to provide policies regarding the autopsies of suspected or confirmed COVID-19 cases.

### Italian interim recommendations – the scientific society of hospital legal medicine of the national health system with Italian society of anatomical pathology and cytology

During the autopsy of an individual with suspected COVID-19, the following PPE should be applied: headgear, a double pair of gloves, cut-resistant protective gloves, FFP3, goggles or a protective visor, a long-sleeved gown or a waterproof suit and overshoes. In a confirmed COVID-19 case, with both clinical and imaging confirmation, either postmortem core biopsies or a discretionary autopsy alone should be performed. When there is a need to collect samples from a body of the deceased with either suspected or confirmed COVID-19, it is also recommended that samples from multiple organs (lungs, liver, and skeletal muscles) be collected applying core biopsy sampling. Furthermore, a multidisciplinary group including at least one clinician and one radiologist should request sample collection. In patients with suspected or probable COVID-19, both nasopharyngeal and oropharyngeal swabs should be collected; if the tests are negative, an autopsy can be performed [28]. In patients without apparent SARS-CoV-2 infection and pulmonary problems and/or complications, several precautions and procedures should also be followed. In such cases, it is crucial to collect oropharyngeal swabs (within 2 h of death) to test for potential SARS-CoV-2 infection; the results should be obtained within 24 h; otherwise, the autopsy cannot be performed. Such precautions are of the utmost importance, as it was reported primarily in Northern Italy that even in patients with a negative SARS-CoV-2 test, several histological findings that are characteristic of COVID-19 were found in such bodies, and these primarily included macroscopic and microscopic alterations within the lungs.

## Viral persistence and sample collection

Knowledge about the persistence of SARS-CoV-2 on different surfaces is limited only to laboratory settings, with no precise confirmation in reality. It is yet unknown how long SARS-CoV-2 can persist on the clothes and bodies of the deceased. It was found that the stability of SARS-CoV-2 is similar to that of SARS-CoV-1; however, this was proven only under experimental conditions [29]. According to Kampf et al., human coronaviruses remain infectious on inanimate surfaces for up to 9 days [30]. Furthermore, it is estimated that coronavirus infectivity can be significantly reduced upon applying 0.1% sodium hypochlorite or 62–71% ethanol to surfaces. Copper also exhibits antiviral properties [31]. However, it is has not yet been confirmed whether the abovementioned findings apply specifically to SARS-CoV-2. Coronaviruses are sensitive to increased temperatures, detergents and commonly used disinfectants [30]. Viral transmission, usually on items via the airborne route, results in viral persistence on those surfaces, usually up to several hours. On cardboard surfaces, viruses can persist up to 24 h, whereas on stainless steel and artificial materials, viral persistence might last up to 2–3 days [29]. Viruses that are closely related to SARS-CoV-2 – SARS and MERS – can persist for a long time in humid and shaded environments – up to 2 days in tap water and sewage, up to 3 days in feces, up to 14 days in saline, and up to 17 days in urine [32]. MERS-CoV might be detected in nasal swabs up to 3 days after death [33]. SARS-CoV-2 can also be detected in cerebrospinal fluid; however, such results are limited to ante mortem studies [34].

The results of available studies show that SARS-CoV-2 detection is possible mainly in secretions from the respiratory tract. The highest positive rates of SARS-CoV-2 detection are noted in bronchoalveolar lavage fluid, followed by sputum, nasal swabs, fibrobronchoscope brush biopsy, pharyngeal swabs, feces, and blood (Table 2) [35–37].

**Table 2:** Recommendations of the Polish Society of Forensic Medicine and Criminology regarding postmortem sample collection

Autopsy material for additional examinations	1 swab from the back of the nasal passages 2 swabs from the lower respiratory tract (from each lung) A standard set of organ pieces (immersed in formaldehyde) for a microscopic examination with an additional collection of samples from the mainstream bronchus Blood sample for a possible biochemical tests Optional collection of other samples or swabs for determining the markers of sepsis or inflammation
Handling with the autopsy material	Swab sticks should be made of plastic with a proper transfer medium dedicated for virological tests (ready-to-use-swabs) Samples should be signed previously to swab collection Samples should be immediately sent to the laboratory; in such cases, a sample should be placed in ice to provide an optimal temperature (5 ± 3 °C). In cases when a sample must be stored for more than 24 h, it should be frozen at a temperature of -70 °C. In such circumstances, the samples should be transported to the laboratory in dry ice

The highest sensitivity of SARS-CoV-2 detection is obtained via the collection of swabs from both the upper and lower respiratory tracts (among which bronchoalveolar lavage fluid and sputum are the most highly recommended) [38, 39]. An autopsy allows the collection of swabs from the lower respiratory tract, which provide a higher probability of viral RNA detection than swabs from the nasopharynx.

Before an anatomical examination and autopsy, all the samples for further testing should be collected to minimize the risk of potential contamination [32]. Generally, there are several rules regarding sample collection:

1. Samples for etiological and electron microscopy testing and cryopreservation should be collected first.
2. Samples for etiological gene testing should be stored in Hank’s solution (nucleic acid-free before use) or transfer medium dedicated to virological tests.
3. Tissues that require freezing can be cut into blocks and stored in plastic bottles.
4. Tissues that require an electron microscopy examination can be cut into blocks and stored in 3% glutaraldehyde.
5. Tissues for conventional paraffin embedding can be stored in a 4% paraformaldehyde solution for approximately 48–72 h before further examination [32].

Samples that are recommended to be obtained during an autopsy include 2 samples from the peripheral blood, 1 sample from the blood containing an anticoagulant for genetic examinations, 1 sample from urine, 1 sample from cerebrospinal fluid, and 2 samples from vitreous humor, as well as pleural, pericardial, or peritoneal fluid, if possible [40, 41]. SARS-CoV-2 might also be detected in the feces of patients who do not present with any gastrointestinal symptoms. Viral detection limited only to oral swabs is not recommended since the virus is detected only in anal or blood samples and is not detected in oral swabs. Furthermore, oral swabs that are positive during the early stages of infection can become anal swabs that are positive during the late stages of infection [42]. Respiratory tract samples can be easily collected with ready-to-use swabs; blood, cerebrospinal fluid, or urine samples should be taken prior to the opening of body cavities to minimize the risk of contamination. Blood cultures can be preferentially obtained from the subclavian vein, jugular vein, or left ventricle [43]. It has been suggested that the pooling of samples before RT-PCR amplification might reduce the number of further tests needed [44]. Cryopreservation allows the rapid and deep freezing of tissues (at a minimum temperature of -80 °C) and thus the collection of samples with viruses for further diagnostic tests. This is highly beneficial, especially where tests are limited and cannot be performed immediately after sample collection. In COVID-19 patients, bronchial tissue samples can be collected for cryopreservation. However, since different samples were reported to be obtained from the deceased so far, it should be taken into consideration that standardized autopsy procedures (including sample collection) are crucial to facilitate a postmortem diagnosis.

### SARS-CoV and MERS-CoV sample collection

The laboratory diagnosis of SARS-CoV includes sample collection from different sites. Tracheal aspirate and stool samples are believed to have the highest detection yield; throat samples pooled with nasal, rectal, and throat swabs and nasopharyngeal aspirate present a moderate detection yield; and throat washing and urine samples present the lowest detection yield [45]. SARS-CoV is also detectable in tears [46].

Regarding MERS-CoV, the specimens include samples from the lower respiratory tract (bronchoalveolar lavage, tracheal aspirate, pleural fluid, and sputum) and upper respiratory tract (nasopharyngeal swab, oropharyngeal swab, and either nasopharyngeal wash or nasopharyngeal or nasal aspirate) [47].

## Autopsy room requirements

The rooms where autopsies are performed should meet specific technical requirements, with a particular emphasis on the ventilation devices. First, two zones should be designated – a 'clear area' and a 'dirty area' – and an additional 'intermediate zone' for the removal of protective clothing after the autopsy [37]. Autopsies should be performed in airborne infection isolation rooms (AIIRs), which should provide negative pressure compared to the surrounding areas [22]. When such rooms are unavailable, negative pressure should be provided, and air recirculation to other rooms should be restricted. It is recommended to provide air exchange (a minimum number of 6 changes per hour); air should be exhausted either outside or through a high-efficiency particulate aerosol (HEPA) filter. Equipment in the autopsy room dedicated for COVID-19 autopsies should include:

- An oscillating or hand-held saw 
- Antiviral disinfectants and specific devices for fumigation decontamination (optimally) 
- A separate table for sample preparation for further examinations 
- Medical waste containers
- Video transmission of the autopsy for the prosecutors (to minimize the number of individuals in the autopsy room) [8, 22]. 

## Potential postmortem tests and examinations

Three major examinations are the most important regarding COVID-19 confirmation: real-time polymerase chain reaction, immunochemistry, and electronic microscopy. Serological tests depend on the so-called ‘serological window’, which, in the case of SARS-CoV-2, lasts for 7 to 14 days [48]. Both molecular and serological tests are crucial to confirm viral presence since SARS-CoV-2 can be transmitted through various routes [49]. Postmortem examinations raise numerous concerns not only in patients with confirmed COVID-19 but also in patients with suspected or probable COVID-19. This is mainly due to limited possibilities of performing extensive population screening tests that exceed logistical and financial capabilities even in highly developed countries. Postmortem examinations and tests for SARS-CoV-2 are highly important, as they enable the maintenance of proper precautions adjusted to the actual threat level not only during the autopsy but also in the further management of the deceased [50]. Given the current epidemiological situation, autopsies, even in patients with unknown or uncertain causes of death, should be performed with extreme precautions following an appropriate preventive policy, as unconfirmed cases create a source of potential contamination [8]. Therefore, it is proposed that computed tomography (CT) might constitute an alternative method of COVID-19 detection and significantly minimize the risk of potential infection in medical personnel. The usefulness of CT has been documented by many ante mortem studies, and its sensitivity is believed to be even higher than that of genetic or immunoenzymatic tests [51–57]. The application of CT is crucial since radiological changes might appear as the first signs, preceding clinical manifestations in some cases. In Italy, a country with one of the highest mortality and incidence rates of SARS-CoV-2 infection, postmortem computed tomography (PMCT) is recommended as a primary diagnostic tool for COVID-19, being equally as important as genetic tests [28]. In addition, PMCT is considered a highly sensitive diagnostic method, especially in patients who did not receive cardiopulmonary ante mortem resuscitation [58]. While performing PMCT in suspected or confirmed COVID-19 cases, it is important to consult a radiologist who specializes in postmortem imaging, as it is crucial to distinguish between naturally occurring postmortem changes in the lungs and those that were induced by SARS-CoV-2 infection and COVID-19 [51, 59]. PMCT might be a useful tool during the autopsy of confirmed and suspected COVID-19 cases; however, the utility of this examination should be further evaluated. In addition, lung ultrasound can be used in children with pneumonia, for instance. However, such findings should be cautiously interpreted with the clinical and laboratory findings [60]. To the best of our knowledge, it has been unknown whether USG can be applied as a postmortem diagnostic tool in patients with SARS-CoV-2 infection. Although there are several preliminary studies on USG as a tool for forensic purposes, knowledge of its usefulness in COVID-19 patients is lacking [61–65]. In addition, specific procedures regarding the handling of corpses require appropriate PPE that minimizes the risk of potential viral transmission (Table 3).

**Table 3:** A list of minimum personal protective equipment required during autopsies in either suspected or confirmed COVID-19 cases

Handling of the corpses	Protective suit	Surgical gown	Interlining apron	N95 + mask	Surgical mask	Glasses / googles	Surgical gloves	Strengthened gloves
Inserting a corpse into a bag	+	-	-	+	-	+	+ / + +	-
Inserting into the second bag	-	-	+	-	+	+	+	-
Transport of corpses in bags	-	-	-	-	-	-	+	-
Taking to the cold room	-	-	+	-	+	-	+	-
Post-mortem CT scan	-	-	+	-	+	-	+	-
Swab collection	-	+	-	±	+	+	+ / + +	-
Peripheral blood collection	-	+	-	±	+	+	+ +	-
External examination of the body	±	+	-	±	+	+	+ +	-
Autopsy	+	-	-	+	-	+	+ +	+

„-”Unnecessary „+” required „++”optimally double „+/-”optional

## Radiological findings

The two most common imaging characteristics of COVID-19 are ground-glass opacities and lung consolidation [66, 67]. Lesions that are found via CT are primarily in the middle and lower lung sites and present a multifocal distribution [56]. Bai et al. showed that the prevalent features of COVID-19 found via CT also include a peripheral distribution of pneumonia (80%) and vascular thickening (58%) [68]. However, CT findings vary in different patients and stages of COVID-19. Pleural thickening and extensive adhesion have been reported in COVID-19 patients, consistent with PMCT and autopsy findings [69]. The most common pulmonary imaging features observed by Zhao et al., who performed CT on 101 individuals with COVID-19, were ground-glass opacities (86%), ground-glass opacities with consolidation (64%). vascular enlargement (71%) and traction bronchiectasis (53%) [70]. Other prevalent findings observed via PMCT include mixed patterns of reticular infiltrations [71]. Such findings are distributed mostly peripherally in the lower lobes and usually involve both lungs. In addition to CT, X-ray can also be performed in COVID-19 patients and demonstrates patchy, high-density opacities or consolidation in the lungs with high accuracy [72]. Conventional chest CT or X-ray is sufficient to identify typical characteristics of COVID-19-associated pneumonia.

Currently, there are no sufficient data on the viability of PMCT as a postmortem diagnostic tool for patients with suspected or confirmed COVID-19. However, according to Italian recommendations, PMCT has been included in the decision tree of the autopsy, and its utility is equal to that of swab collection [28]. The sensitivity and specificity of this method are yet unknown in postmortem studies. However, if there is a limited number of diagnostic tests, PMCT and radiological findings might constitute a solution that could minimize the risk of infection. However, there is a need for further validation studies to verify the utility of PMCT application during the autopsies of patients with suspected/confirmed COVID-19. Importantly, even in asymptomatic patients with yet unconfirmed or suspected COVID-19 pneumonia, CT can reveal typical abnormalities [67].

## Pathological postmortem findings

### Macroscopic manifestations

An autopsy requires the confirmation of SARS-CoV-2 infection, usually via sample collection and further rRT-PCR tests. The majority of macroscopic features of COVID-19 are observed primarily within the chest cavity, and the most common findings include lung consolidation, pulmonary edema, pleurisy, or pericarditis; additionally, the weight of the lungs might be increased [8, 43]. Macroscopically, lung parenchyma might be edematous and firm, which is consistent with clinical findings common for acute respiratory distress syndrome [73]. Liu et al. described an 85-year-old Chinese patient who died because of COVID-19, and the gross autopsy showed that the lungs were heavy, with a significant amount of gray-white viscous fluid; at the same time, other organs, such as the heart, liver, and kidneys, remained unchanged [74]. Konopka et al. described an asthmatic patient with COVID-19; the gross postmortem examination showed an increased weight of the lungs with mucus plugging and consolidation of lung parenchyma [75]. A prevalent postmortem finding of individuals with COVID-19 includes the presence of mucus in the airways; furthermore, the lungs are reported to be heavy, edematous, and relatively firm in texture [76]. It is crucial to perform a lung autopsy very scrupulously since in some cases, negative nasopharyngeal swabs do not exclude the possibility of SARS-CoV-2 in lung parenchyma. Conversely, lung parenchymal swabs might remain negative while nasopharyngeal swabs can become positive for SARS-CoV-2 at the same time.

### Microscopic manifestations

Edler et al. described a cohort of 34 deceased females and 46 males who died because of multiorgan failure related to COVID-19 [77]. The histological findings observed during the lung tissue autopsy revealed alveolar spaces with a focal hyaline membrane, pneumocyte proliferation and metaplastic changes, in some of the cases, especially in the advanced stages fibrosis was observed. Lymphocytes and plasma cells were the most prevalent inflammatory cells present within the pathologically changed lung tissue. Elsoukkary et al. showed that most of the deceased patients presented both—the exudative and proliferative alveolar damage with the excessive amounts of neutrophils and pneumocytes type II with reactive atypia and bronchial squamous metaplasia [78]. Viral cytopathic effects and a slight increase in alveolar wall thickness are changes typical of viral pneumonia, whereas the presence of hyaline membranes is common in acute respiratory distress syndrome. Acute respiratory distress syndrome is characterized by the presence of hyaline membranes in the alveoli during the acute stage, whereas the organizing stage is characterized by interstitial widening by edema, as well as fibroblast proliferation [79]. Postmortem histological findings also include diffuse alveolar damage, such as a disrupted alveolar septa, the proliferation and desquamation of type II alveolar epithelial cells, the exudation of fibrin, monocytes, and macrophages, capillary congestion, and hyaline membrane formation [80–83]. Diffuse alveolar damage is highly prevalent among COVID-19 patients in whom acute respiratory distress syndrome is diagnosed ante mortem. The abovementioned histological findings are similar to those observed during the autopsy of patients who died because of SARS or MERS [84–86].

**Fig. 1:**
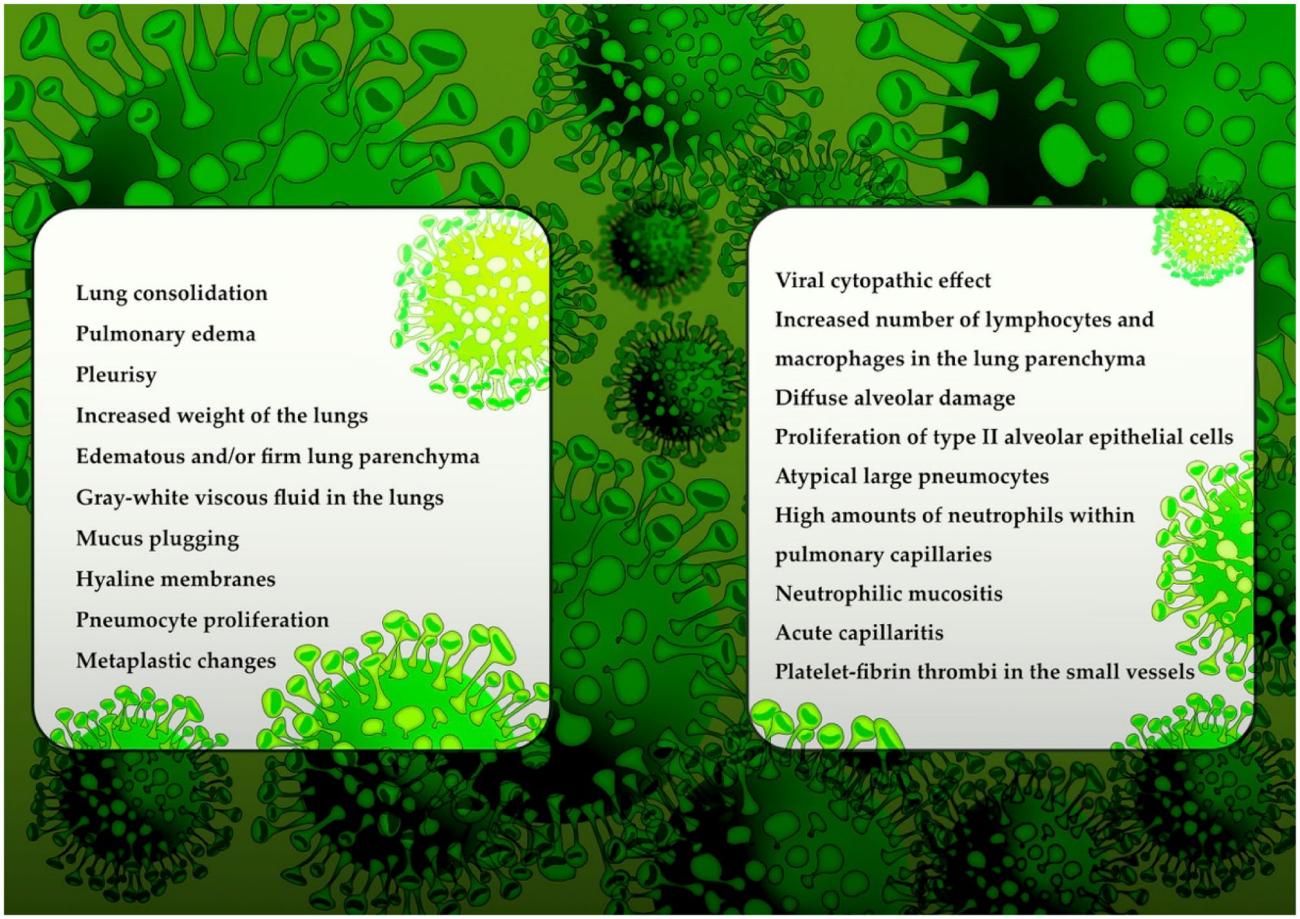
Postmortem macroscopic and microscopic findings in COVID-19 patients

Several nonspecific manifestations typical of viral infections include edema, hyperplastic pneumocytes, multinucleated giant cells, proteinaceous/fibrinous exudates or patchy inflammation [87]. In some cases, lung parenchyma might not be macroscopically changed, even though histopathologic biopsies might show pathologies such as diffuse alveolar damage, signs typical of acute respiratory distress syndrome, interstitial lymphocytic infiltrates, atypical large pneumocytes, or cytopathic changes [84, 88]. In addition to diffuse alveolar damage, chronic inflammation and edema in the bronchial mucosa can also be observed during the autopsy (Fig. 1) [89].

The lung parenchyma of patients with COVID-19 might contain inflammatory components characterized by numerous CD68-positive macrophages and only a few CD45 lymphocytes that are found primarily in the interstitial space [90]. Lung samples collected from patients with COVID-19 usually contain large numbers of neutrophils within pulmonary capillaries; neutrophilic mucositis and acute capillaritis with enhanced fibrin deposition have also been described [91]. In addition, it is suggested that enhanced neutrophilia is associated with an excess of neutrophil extracellular traps. Another prevalent finding is the presence of platelet–fibrin thrombi in the small vessels that exhibit a high prevalence of coagulopathy among COVID-19 patients. Postmortem microscopic findings in COVID-19 patients might indicate bronchopneumonia [75]. Su et al. also reported renal histopathological alterations in COVID-19 patients with occlusion of the microvascular lumina by erythrocytes, endothelial damage, and glomerular and vascular changes [92].

## Burials during COVID-19 pandemic

Patients who die because of COVID-19 can be embalmed, buried or cremated depending on the preferences [93]. As people from different religions have numerous traditions, the burial, cremation, or embalming tends to differ. Embalming is the injection of a preservative solution that replaces the blood and fills the body cavities and organs [94]. Since embalming involves contact with the body, blood, and other fluids, this process should be undertaken with extreme precautions to minimize the risk of infection.

The Catholic Cemeteries & Funeral Services (CCFS) prepared protocols to handle individuals who died because of COVID-19 that were adjusted for specific situations; mortuary services have continued without interruption in Catholic Church, and the procedures might involve traditional burial, cremation or embalming [95]. The European Centre for Disease Prevention and Control (ECDC) allows burial, cremation, or embalming of the deceased with either suspected or confirmed COVID-19 [93]. The ECDC states that the families are allowed to view the body. Additionally, permission is granted for physical contact with the body; however, following standard precautions and applying PPE – gloves and long-sleeved water-resistant gowns are the minimum requirements. In cases of a PPE shortage, physical contact with the body should be restricted. According to Red Cross guidelines, burial in a single grave is preferred, whereas mass graves are discouraged; the body should also be double bagged [96]. In Jewish culture, regardless of the burial method performed, the seventh day following a burial – shiva – is the day at which the grieving process begins and is very important in Jewish culture; however, due to the need for social distancing and gathering prevention – it has to be canceled or postponed [97, 98]. In addition, according to Jewish religious law, burial must be undertaken within 48 h of death, which might be naturally restricted during the SARS-CoV-2 pandemic. In Islamic law and Muslim culture, each individual should be buried in a separate grave; however, in cases of a massive SARS-CoV-2 infection, collective graves are permitted by the law [99]. Buddhists perceive an autopsy as beneficial; however, they usually prefer to prolong the examination for 3–4 days to ‘give time for the soul to leave the body’; during the COVID-19 pandemic, these timelines have to be shortened [100].

Generally, families and friends may see the body before the burial; however, the restrictions concern physical contact. In addition, under the principles of social distancing, the number of mourners must be limited. Individuals who have certain respiratory diseases should not be allowed to see the body; however, it is possible only if the person wears a protective mask. Furthermore, children, adults over 60 years old, and immunosuppressed individuals should not have contact with the body. During all the activities associated with body preparation for burial, the usual principles of Standard Infection Control Precautions and Transmission-based Precautions should be followed. The risk assessment regarding the funeral premises should include known or suspected infection hazards, the timing of procedures, the number of required staff, and whether additional observers should be excluded [9]. Burial procedures are not systematized, but they should be since the lack of proper and sufficient protective precautions might be associated with an increased epidemiological risk.

## Conclusions

SARS-CoV-2 has been reported to present certain survivability in the bodies of infected patients. There are still speculations as to whether individuals with confirmed COVID-19 should receive an autopsy. However, during times of rapid pandemic expansion and the urgent need for epidemic preservation, the need for an autopsy in both suspected and confirmed cases seems rational since it expands our knowledge of the mechanism of action of SARS-CoV-2 and organs that are targeted by the virus. An autopsy allows the exact macroscopic and microscopic examinations of suspected and confirmed cases. In addition, an autopsy provides an accurate diagnosis and confirms ante mortem laboratory and radiological findings, especially in suspected cases. Furthermore, an autopsy allows the detection of coexisting diseases as well as the assessment of the extent of SARS-CoV-2 infection. Although there have been an increasing number of studies regarding the clinical manifestations and complications of COVID-19, knowledge about the pathologic postmortem manifestations, both macroscopic and microscopic, is still scarce. Since the pandemic is still expanding and the number of infected patients is continually increasing, there is an urgent need to report postmortem findings to expand our knowledge on the pathology of COVID-19. To increase the efficiency of work while minimizing the risk of exposure of medical personnel to the potential source of infection, it is highly recommended to introduce PMCT on a regular basis while simultaneously performing needle biopsy to collect lung parenchyma specimens instead of opening all body cavities. For forensic experts and pathologists, it seems necessary to collect different samples because viral detection might differ depending on the stage of the disease. Minimally invasive biopsy for a postmortem histological examination and the application of a virtual autopsy (so-called virtopsy) might constitute alternatives for a typical autopsy, significantly minimizing the risk of potential infection.

## Key points

1. Severe acute respiratory syndrome coronavirus 2 (SARS-CoV-2) is characterized by survivability in the bodies of the deceased who died because of SARS-CoV-2 infection.

2. Autopsies are rational for patients with both suspected and confirmed SARS-CoV-2 infection.

3. Postmortem findings of SARS-CoV-2 infection include macroscopic and microscopic changes, as well as altered radiological findings.

4. SARS-CoV-2 might be detected in various samples obtained postmortem, such as respiratory tract samples, blood, feces, cerebrospinal fluid, or urine.
